# Environmental filtering of body size and darker coloration in pollinator communities indicate thermal restrictions on bees, but not flies, at high elevations

**DOI:** 10.7717/peerj.7867

**Published:** 2019-10-14

**Authors:** Lindsie M. McCabe, Neil S. Cobb, Bradley J. Butterfield

**Affiliations:** Merriam-Powell Center for Environmental Research and Department of Biological Sciences, Northern Arizona University, Flagstaff, AZ, United States of America

**Keywords:** Pollinators, Elevational gradients, Bees, Flies, Functional traits, Body size, Body darkness

## Abstract

**Background:**

Bees and flies are the two most dominant pollinator taxa in mountain environments of the Southwest USA. Communities of both taxa change dramatically along elevation gradients. We examined whether bee and fly traits would also change along elevation gradients and if so, do they change in a predictable way related to a decrease in temperature as elevation increases.

**Methods:**

We used insect body size and darkness traits as proxies for energetic requirements and indicators of cold tolerance in order to assess patterns of bee and fly community trait differences along an elevation gradient. We examined 1,922 individuals of bees and flies sampled along an elevation gradient ranging from 2,400 meters to 3,200 meters and from 9.6 °C to 5.2 °C mean annual temperature. We examined bees and flies separately using community weighted means (site-level trait values weighted by species abundance) and estimates of environmental filtering (quantified as the inverse of the standardized range of trait values).

**Results:**

Bees and flies exhibited two somewhat distinct patterns; (1) Community weighted mean body volume and darkness of bees increased sharply at the highest elevation, and the intensity of environmental filtering also increased with elevation. This was due to both a change among bee populations within a species as well as species replacement at the highest elevation. (2) Community weighted mean body volume and darkness of flies also increased moderately with increasing elevation, but did not exhibit patterns of significant environmental filtering. In fact, the intensity of environmental filtering as indicated by the range of fly body volume weakened with elevation.

**Conclusion:**

The increase in filter intensity at high elevations exhibited by bees suggests a significant limitation on the breadth of viable functional strategies for coping with extreme cold, at least within this regional species pool. Flies, on the other hand, do not appear to be limited by high elevations, indicating that the shift from bee to fly dominance at high elevations may be due, at least in part, to greater environmental constraints on bee adaptation to colder environments.

## Introduction

High-elevations generally represent restrictive environments in which only a narrow range of ecological adaptations are viable. Rahbek (1995) reviewed 163 ecological studies and confirmed that species richness decreases as elevation increases in almost every taxonomic group, including plants (Keller, Kienast & Beniston, 2000), animals (Terborgh, 1977), and even aquatic bacteria (Zeng et al., 2016). Environmental filtering can have a large effect on community composition in high-elevation environments by excluding individuals that are not adapted to stressful mountain top environments, which usually include cold temperatures and short growing seasons (Körner, 2003). Elucidating patterns of environmental filtering will help us understand the distribution of biodiversity along elevation gradients and may help us to predict the impacts of less restrictive environmental filters associated with increasing temperatures.

Variation in functional traits (i.e., organismal characteristics that mediate responses to the environment) among species within communities can provide insight into processes of community assemblage (Lavorel & Garnier, 2002). The breadth of trait values within a community can be indicative of the intensity of environmental filtering, where a narrow range of trait values indicates strong environmental filtering (Cornwell & Ackerly, 2009; Kraft & Ackerly, 2010; Messier, McGill & Lechowicz, 2010). Following the “stress-dominance hypothesis” (Coyle et al., 2014), the breadth of trait values within communities is expected to decrease in high elevation environments due to the abiotically-limiting environments. Furthermore, variation in community-weighted mean trait values along elevation gradients can identify shifts in realized morphological trajectories in adaptation. Taken together, variation in the range and central tendency of trait distributions along elevation gradients can reveal shifts in both the direction and intensity of environmental filtering.

Functional traits associated with low temperature tolerance strongly influence adaptation to high elevation environments (Körner, 2003). Hodkinson (2005) proposed that insects with greater body size and/or darker body color could be better adapted to the lower temperatures of high elevation environments. Darker coloration increases heat absorption potential which in turn will raise the thoracic temperature of insects (Heinrich, 1996; Hodkinson, 2005), and greater body size improves the ability for species to thermoregulate their thoracic wing muscles (Mayr, 1956). Traits such as body darkness and body size may also differentially affect taxa based on their fitness costs. For example, lower environmental temperatures may more acutely affect active insects, such as bees, which expend a great deal of energy in foraging for their offspring (Heinrich, 1975), whereas flies do not require pollen or nectar resources for their offspring and require less pollen resources for themselves than do foraging adult bees (Haslett, 1989). Due to differing maternal requirements of these two taxa, such as bees having to collect nectar/pollen resources for their offspring, functional traits could be used to assess the differences in environmental filtering within these two groups.

In this paper, we assess functional trait variation within and among bee and fly communities and species to test the overarching hypothesis that environmental filtering intensifies with increasing elevation. Specifically, we tested the hypotheses that (1) a larger body size and darker coloration of the bee’s and fly’s body will increase with elevation, and (2) the range of trait values within communities, relative to expected values based on a null model of community assembly, will become narrower with increasing elevation and we will see an environmental filtering effect at high elevation environments. We predicted that these patterns will be stronger for bees than for flies, given the greater species diversity, habitat affiliation (McCabe et al., 2019) and stronger ties to their host plants while flies tend to be more generalized with host plant selection (Lefebvre et al., 2018) Assessing trait patterns can provide insight into the constraints of temperature on the composition of high elevation pollinator communities.

## Methods

### Sampling

Sampling was conducted on the C. Hart Merriam Elevation Gradient along the North side of the San Francisco Peaks near Flagstaff, Arizona. This gradient was the inspiration for the development of the life-zone concept (Merriam, 1890), and has been a model system for understanding variation in ecological processes along an elevation gradient (Wu et al., 2012). We focused on three higher life zones of this gradient; ponderosa pine (∼2,400 m), mixed conifer (∼2,600 m) and spruce-fir (∼3,200 m) forests, because this was the presumed transition from bee dominated communities to fly dominated communities. Samples for this study were taken from a larger study documenting the transition from bee dominated pollinator communities at lower elevations to fly dominated communities at higher elevations (McCabe et al., 2019). Three sites were established at each of the three life zones, all at least 2 km apart. Each site included two habitats: a forest and a meadow. Meadow traps were placed in the middle of the meadow, at least 50 m from the edge of the road. Forest traps were placed 100 m from the edge of the meadow. At each site, we placed a pollinator cup (i.e., elevated pan trap) array, which consisted of twelve 59 ml plastic cups (4 white, 4 yellow and 4 blue). Each cup was filled with a 50:50 mixture of water and food-grade propylene glycol. A total of 108 cups were deployed during the dry pre-monsoon (June 4–7, 2013) and 108 pollinator cups during the monsoon (August 13–16, 2013) seasons. This sampling method accounted for species that are active in early spring after snow melt as well as during the monsoon period, when the majority of the wild flowers in Northern Arizona bloom (Smith et al., 2015). Each array consisted of 12 pollinator cups; each cup was 3 m from one another and placed in “Y” shape with each color on a line. We chose to use elevated “pan traps” because previous studies in our area (Smith et al., 2015) as well as other areas in the southwest have found that ground level pan traps do not represent the true diversity of bees in that community (Cane, Minckley & Kervin, 2001). Our traps were set at 25 cm above ground level, which we found to be the average height of all herbaceous plants along the elevation gradient. All insects were collected using propylene glycol solution and then washed and dried in the same manner and timeframe to prevent matted hairs or swelling of the abdomen. Finally, all specimens were properly curated and pinned straight to avoid distortion due to the position of the specimen.

### Trait measurements

We analyzed a total of 1,922 specimens, 1,283 bee and 639 flies. This included 96 species of flies and 178 species of bees. Fly pollinators were dominated by species from Tachinidae and Syrphidae, with few species from Bombyliidae and the superfamily Muscoidea. Bees species found along the gradient encompassed all five dominant bee families found in North America, with a high species variability between life zones.

Images were taken on a BK Plus Lab System from Visionary Digital using a Canon EOS 5D Mark II camera and 65 mm lens. All sliced images per specimen were taken in RAW and then for each specimen images were stacked for full focus of the final image. Final images were saved as a 16-bit TIFF and consisted of 12–35 stacked images depending on the size of the specimen. Final processing of the images included a color correction to an 11% neutral gray background which matched the specimen plates used in imaging all specimens. Neutral gray was used as a mid-point between black and white, where the light that reflects back is a neutral gray, rather than blue or red tones. Using the standardized color match lets us accurately project the colors of each specimen. All images were taken using two twin 250 watt halogen modeling lamps and a 2,000 watt-second Xenon Flash. Three composite images were taken of each specimen: a dorsal, lateral, and anterior image. Volume was measured as an ellipsoid area based on length (dorsal image), width (dorsal) and height (lateral). Head volume was not included in the measurements, because the measurement can vary based on the position and angle of the head when collected. With the same images used for measuring body volume, body darkness was calculated as the median saturation value using the ImageJ histogram function. Saturation values range from 0 to 222, with 0 representing an all-white image and 222 representing an all-black image. In each image, the face, thorax and abdomen, with wings and legs excluded, were selected to estimate body darkness. We used ImageJ 1.46r (2014) to quantify body volume and darkness of all bees and flies caught in the pollinator cups.

### Functional composition and diversity

We calculated both the community-weighted mean (CWM) and range of trait values across all individuals within each sample (pollinator cup) using the ‘FD’ package in R (Laliberté, Legendre & Shipley, 2014). These values were calculated separately for bee and fly assemblages. Additionally, we calculated the standardized effect size of trait ranges (sesRange), which provides an estimate of habitat filtering that controls for variation in species richness (Cornwell & Ackerly, 2009). The sesRange is determined by comparing the observed range of trait values in a sample to a null distribution of expected values, which was generated for each level of observed individual richness by randomly drawing from the regional pool of individuals without replacement 1,000 times. The sesRange was calculated as sesRange = (obsRange–nullRange)/nullRange, where obsRange is the observed range of trait values in a sample, and nullRange is the mean range estimate of the null distribution with the total community as the observed sample. Positive values of sesRange are interpreted as representing weak environmental filters, and negative values as strong environmental filters, with values ≥—1.96— (2 standard deviations) significantly different from the null expectation with two-tailed *α* = 0.05.

### Statistical analysis

A one-way analysis of variance (ANOVA) was used to predict variation in CWM and sesRange of bee volume, bee darkness, fly volume and fly darkness (8 ANOVAs total), using life zone (ponderosa, mixed conifer and spruce-fir) only as predictor variables. Tukey’s post-hoc tests were then performed as a post hoc test. Pollinator cups at each site and season were combined in this analysis because we were primarily interested in the change along the elevation gradient and not trap color preference or seasonal differences. A prior analysis (ANOVA & mixed effects model) was run to test the effects of habitat on CWM and sesRange of bee volume, bee darkness, fly volume and fly darkness, however no effects were found for any of the eight response variables, therefore this analysis was excluded (SI.1). An additional analysis was run using a mixed effects model to compare site as a random effect and life zone as a predictor variable. However, no differences were seen between the ANOVA and mixed effects model (SI.2), based on AIC scores, therefore we used the analysis with the more interpretable approach. One-way ANOVAs were also used to analyze intraspecific variation of trait values along the elevation gradient. All data analyses were conducted using R 3.3.1 (R Core Team, 2013). Body darkness and body volume were independent of one another at the individual insect level, both in flies (*r* =  − 0.087; *p* = 0.447) and bees (*r* = 0.263; *p* = 0.365), thus both traits were analyzed independently.

We performed a non-metric multidimensional scaling ordination (NMDS) using a Bray-Curtis distance matrix to visualize differences in community composition along the elevational gradient. Analyses were done using R.3.1.2 (R Core Team, 2013) and packages vegan (Oksanen et al., 2017) and ecodist (Goslee & Urban, 2007).

## Results

### Darkness

**Figure 1:**
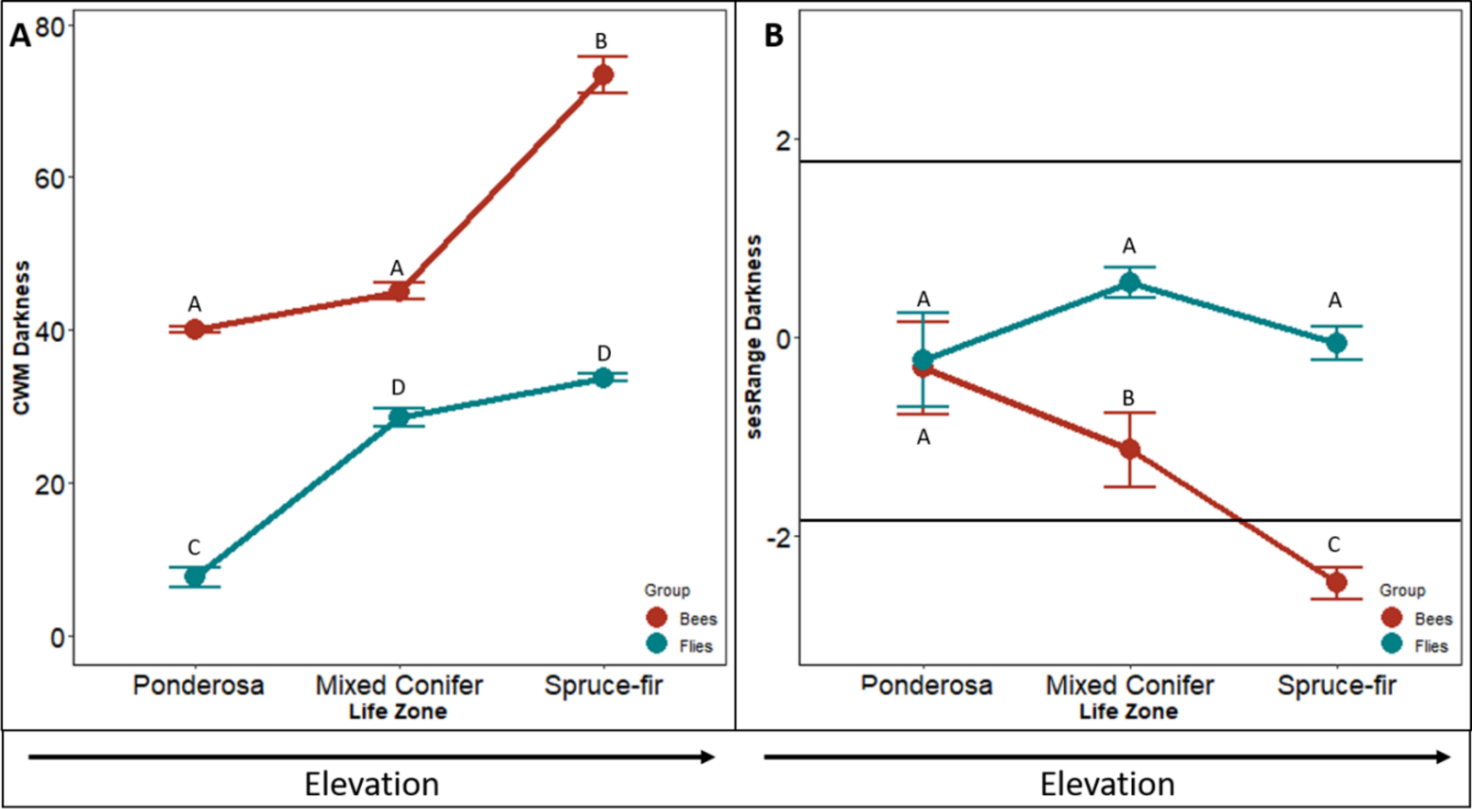
Differences in CWM and SES of body darkness. Differences in community-weighted mean (A) and standardized effect size (B) of body darkness between the three life zones (ponderosa, mixed conifer, and spruce-fir). In (B), values above and below 1.98 and −1.98 (2 SD), respectively, denote a significant standardized effects size with a two-tailed *α* = 0.05. Significant differences based on Tukey’s HSD are indicated by different letters. Data are means with SD.

The community weighted mean (CWM) of bee body darkness increased with increasing elevation (*F*_2,4_ = 931.62, *p* < 0.001). The CWM increased slightly from ponderosa to mixed conifer life zone (*p* = 0.016: forest, *p* = 0.012: meadow) and nearly tripled from mixed-conifer to spruce-fir life zones (*p* < 0.001, Fig. 1A). Species in ponderosa and mixed conifer communities, on average, had a body darkness value of 35, whereas species in spruce-fir, on average, had a body darkness value of 78 (*F*_2,4__=_ 9.34, *p* = 0.006, Fig. 1A).

The sesRange of bee body darkness decreased significantly between each life zone with increasing elevation (*F*_2,4_ = 12.737, *p* < 0.001, Fig. 1B). However, communities did not exhibit an environmental filtering effect until the spruce-fir life zone. The spruce-fir communities had significantly narrower ranges of bee body darkness than expected from the null model (i.e., sesRange <−2 std. dev., sesRange = 0–2.12, *p* = 0.043)).

The CWM of fly body darkness also changed along the elevation gradient, where communities at higher elevations, on average, were larger than species at lower elevations (*F*_2,4_ = 30.844, *p* < 0.001, Fig. 1A). However, unlike the bee communities along the gradient, the greatest change in fly body darkness occurred between ponderosa and mixed conifer (*p* < 0.001), while there was little change between mixed conifer and spruce-fir (*p* = 0.090). Body darkness doubled from ponderosa to mixed conifer, with the average darkness in ponderosa being 14.90, and the average darkness in mixed conifer being 31.21. A similar body darkness between mixed-conifer and spruce-fir was found, with spruce-fir having an average body darkness of 34.69.

The sesRange of fly body darkness was not significantly affected by an increase in elevation (*F*_24_ = 20.883, *p* = 0.587, Fig. 1D). The sesRange of fly body darkness did not change significantly from ponderosa to mixed conifer (*p* = 0.347) nor from mixed-conifer to spruce-fir (*p* = 0.953 Fig. 1D). No life zone showed an environmental filtering effect (ses Range = 0–2.12, *p* = 0.043). There was no effect of niche filtering for any of the life zones (PP = −0.727, MC = 0.420, SF = 0.379).

### Volume

**Figure 2:**
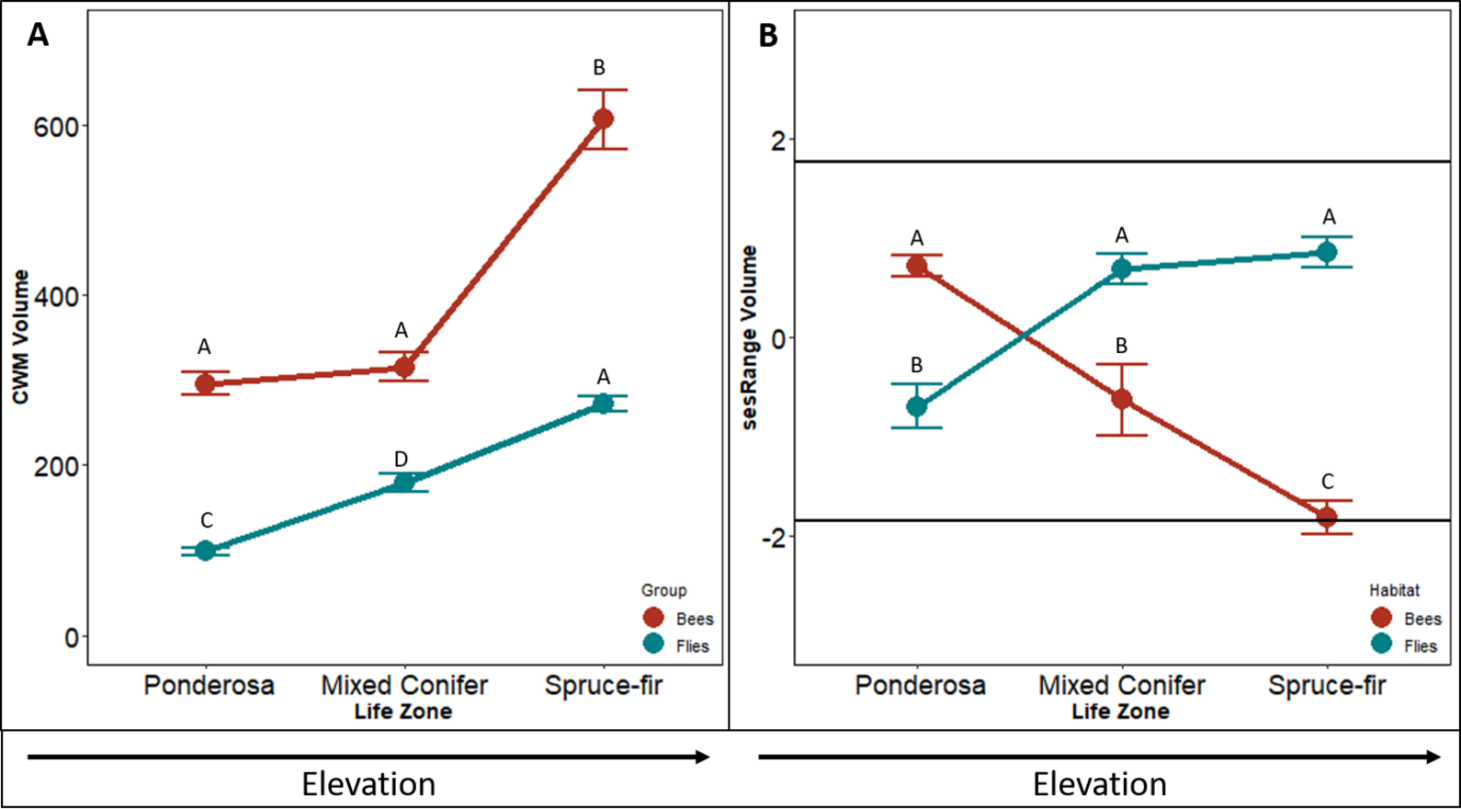
Differences in CWM and SES for body volume. Differences in community-weighted mean (A) and standardized effect size (B) of body volume between the three life zones (ponderosa, mixed conifer, and spruce-fir). In (B), values above and below 1.98 and −1.98 (2 SD), respectively, denote a significant standardized effects size with a two-tailed *α* = 0.05. Significant differences based on Tukey’s HSD are indicated by different letters. Data are means with SD.

The CWM of bee body volume increases along the elevation gradient (*F*_2,4_ = 46.076, *p* < 0.001, Fig. 2A). In similar fashion to the CWM bee body darkness, the CWM of bee body volume increased with a slight increase between ponderosa and mixed-conifer life zones (*p* = 0.156), and a doubling increase in size between the mixed conifer and spruce-fir life zones (*p* < 0.001). On average communities in ponderosa and mixed conifer had a volume on 302 mm^3^ and in spruce-fir the volume average was 598 mm^3^.

The sesRange of bee body volume decreased along the elevation gradient (*F*_2,4_ = 12.737 *p* < 0.001, Fig. 2C). However, no environmental filtering effect was detected until the highest life zone spruce-fir. The sesRange decreased significantly from ponderosa to mixed-conifer (*p* = 0.024), and from mixed-conifer to spruce-fir life zones (sesRange = 0–2.12, *p* = 0.043). Spruce-fir communities had significantly narrower ranges of bee body volumes than expected from the null model.

The CWM of fly body volume increased linearly along the gradient (*F*_2,4_ = 106.66, *p* < 0.001, Fig. 2B). CMW means of fly body volume increased by ∼100mm^3^ from ponderosa to mixed conifer (*p* < 0.001) and from mixed conifer to spruce-fir ( *p* = 0.307, Fig. 2C). Unlike our three other tests of CWM (bee body darkness, bee body volume and fly body darkness) fly body volume did not level off; all life zone measures increased at the same rate. On average, CWM of fly body volume was 100 mm^3^ at ponderosa, 145 mm^3^ at mixed conifer, and 286 mm^3^ at spruce-fir, for both meadow and forest habitats.

Contrary to what we predicted, sesRange of fly body volume actually increased along an elevation gradient, not decreased ( *F*_2,4_ = 20.883, *p* < 0.001, Fig. 2D). The sesRange of fly body volume significantly increased from −0.82 at the ponderosa life zone to 0.86 at the mixed conifer life zone, but did not change from mixed-conifer to spruce-fir. While no significant environmental filtering effect was detected at any life zone along the gradient, the life zones in general got less restricted as elevation increased and not more restrictive.

Ordination analysis showed distinct community differences between both bee and fly communities along the gradient. Bees showed increasingly different communities as elevation increased.

### Intraspecific variation

Variation in CWM trait values were driven by both interspecific and intraspecific variation. For bees, all eight species that occurred at each of the three life zones showed a significant increase of either body volume or body darkness (Table 1). *Bombus occidentalis* (*p* = 0.059) showed a marginally non-significant increase while *Dialictus* 003 (*p* = 0.003), *Heriades* 002 (*p* < 0.001), *Agapostemon texanus* (*p* = 0.029), and *Lithurgus apicalis* (*p* = 0.015) all showed significant increases in body darkness as elevation increased. *Bombus huntii* (*p* = 0.009), *Lasioglossum egregium* (*p* = 0.008), and *Megachile fidelis* (*p* = 0.019) exhibited significant increases in body volume as elevation increased. For flies, only two of five most common species showed an increase in intraspecific trait values as elevation increased. *Xanthoepalpus bicolor* showed an increase in body darkness (*p* < 0.001) and body volume (*p* = 0.008), and *Archytas* 003 showed an increase in body volume (*p* = 0.007), as elevation increased.

**Table 1:** Intraspecific variation of body volume and body darkness for the five most common fly species. Intraspecific variation of body volume (mm^3^) (A) and body darkness (B) for the five most common fly species that persist at each life zone along the gradient and the eight bee species that occupy all three life zones along the gradient. + or − denote a significant change in trait, and in what direction the trait changed, along the elevation gradient.

	**Species**	**Ponderosa**	**Mixed Conifer**	**Spruce-fir**	***df***	***F***	***p*-value**	****
**A:Darkness**								
Flies	*Hemipenthes sinuosa*	47.86	51.47	54.21	2	23.74	0.014	
	*Cynomya cadaverina*	59.77	68.15	71.66	2	1.948	0.287	
	Archytas003	74.74	77.18	63.12	2	0.903	0.425	
	*Tachina Protodejeania*	66.03	69.8	65.16	2	0.899	0.456	
	*Xanthoepalpus bicolor*	71.39	72.91	88.79	2	182.9	<0.001	+
Bees	*Bombus occidentalis*	65.84	79.69	89.88	2	8.205	0.059	+
	*Bombus huntii*	56.61	71.98	86.97	2	3.729	0.154	
	*Dialictus003*	29.19	58.51	90.99	2	16.93	0.003	+
	*Heriades002*	50.62	57.65	70.53	2	19.38	<0.001	+
	*Agapostemon texecana*	68.48	77.84	98.68	2	14.81	0.029	+
	*Lasioglossum egregium*	82.61	82.92	69.1	2	0.207	0.819	
	*Megachile fidelis*	62.62	66.48	70.07	2	5.25	0.105	
	*Lithurgus apicalis*	27.97	35.96	77.65	2	8.98	0.015	+
**B:Volume**								
Flies	*Hemipenthes sinuosa*	48.61	94.11	106.41	2	1.03	0.456	
	*Cynomya cadaverina*	189.25	178.21	273.23	2	0.455	0.672	
	Archytas003	255.93	217.27	149.13	2	3.021	0.077	–
	*Tachina Protodejeania*	290.26	381.91	489.09	2	1.738	0.254	
	*Xanthoepalpus bicolor*	243.61	373.75	975.92	2	35.72	0.008	+
Bees	*Bombus occidentalis*	715.43	2475.98	1781.22	2	0.887	0.501	
	*Bombus huntii*	978.99	1378.25	2613.24	2	5.915	0.009	+
	*Dialictus003*	9.97	17.38	18.56	2	1.269	0.347	
	*Heriades002*	502.04	475.91	377.85	2	0.298	0.749	
	*Agapostemon texecana*	110.02	1124.53	216.57	2	2.577	0.233	
	*Lasioglossum egregium*	100.15	160.69	210.82	2	11.81	0.008	+
	*Megachile fidelis*	386.42	388.41	519.76	2	19.48	0.019	+
	*Lithurgus apicalis*	27.02	26.54	50.03	2	0.44	0.661	

## Discussion

The distribution of morphological traits (body size and body darkness) along a 1,000 meter elevation gradient supports the notion that bee community membership becomes limited in high elevation habitats likely due to thermal constraints. Fly communities, however, did not exhibit a reduction in the range of viable trait values at the same high elevation habitats, despite an increase in both body size and body darkness with increasing elevation, suggesting that they may not be as limited by temperature as bees. The increase in community weighted means along the gradient could suggest that flies benefit for being larger, however there is not enough of a selection pressure in the community to exclude members that are not the largest at high elevations. Similar studies have shown that bee size increases along an elevation gradient (Classen et al., 2017; Hoiss et al., 2012), but no studies have examined how the diversity of these traits, body darkness and body size, may reveal constraints on the composition of pollinator communities, and no study has documented how body darkness in either bees or flies indicate changes in community assembly. Although we focused only on body volume and body darkness, other morphological traits such as setae length (Peat et al., 2005), wing size (Dillon, Frazier & Dudley, 2006), wingbeat patterns (Hodkinson, 2005), and endothermic capacity (Verdú & Lobo, 2008) could also reveal adaptations to, and constraints imposed by, the environment. In the future, all of these characteristics should be considered to more fully understand how community membership is structured at high elevations.

Community-weighted mean body size and darkness both increased with elevation among both groups of pollinators. Flies showed a linear increase in body volume from ponderosa to spruce-fir elevation. Bees, however, did not show a significant increase in community weighted means of either trait until the spruce-fir life zone. High elevation bee species communities are limited and the bee community is primarily dominated by bumblebee species and Megachilidae species at our highest elevation, spruce-fir. Bumblebee species are among the largest bee species and are generally regarded as being high elevation species (Pyke, 1982), and members of the Megachilidae family may also be restricted to high elevations due to their nesting requirements for dead and down trees (Cane, Griswold & Parker, 2007) rather than soil like the majority of other bee species. While we see this strong trend in CWM, we also recognize that there are limitations to the CWM analysis, where the few large species (i.e., bumblebees at high elevations) may be driving the pattern. However, when bumble bees were excluded from the analysis the same patterns still persisted. Megachilidae species may also have limitations outside of temperature such as strong ties to wood nesting resources. This could drive the community patterns since Megachilidae tend to be larger bee species. In general, bee species richness often decreases dramatically with increasing elevation (Inouye & Pike, 1988; Kearns, 1992; Lefebvre et al., 2018; McCabe et al., 2019; McCall & Primack, 1992; Primack, 1978). It has been suggested that the shift from bee dominated pollinator communities to fly dominated communities is primarily due to a greater temperature limitation in bees versus flies (Stone & Willmer, 1989). Thermal-related traits such as body darkness and body volume corroborate what has been proposed previously, specifically the notion that solitary bees are more sensitive to lower temperatures than other pollinating taxa and social bee species (Lee, 2012). Other traits such as the life history characteristics and shorter growing season of essential resources could further reinforce such restrictions on high elevation bee communities. The relative temperature sensitivity of bees compared to flies is even more evident when comparing the standardized ranges of trait values among these communities. For fly communities, no life zone showed a significant restriction in the breadth of viable functional traits values relative to the broader species pool. Bees, on the other hand, showed restricted ranges of trait values at the highest elevation, spruce-fir, for both body volume and body darkness. Differences in thermoregulation and behavior can account for why body volume and body darkness would limit bees but not flies. Insects are mostly ectothermic and do not internally maintain their body temperature, instead relying on the ambient temperature to heat their body (Triplehorn & Johnson, 2005). A larger body size in bees is adapted to lower temperature because they are better able to regulate their internal temperature with warm-up behaviors (Heinrich, 1974; Hodkinson, 2005; Kingsolver, 1983). While flies can wait for the ambient temperature to be warm enough to take flight, the resources needed for bees are quite different and bees therefore must maximize their flight time. For bees, much of their energy is focused on collecting floral resources (i.e., pollen and nectar) for their offspring (Michener, 2007). Flies on the other hand do not require maternal care and therefore only need to collect food for themselves and only long enough to mate (Larson, Kevan & Inouye, 2001). There is increasing evidence to show that species are extending their range to higher elevations linked to global warming (Parmesan, 2006; Stevens, 1992). Assuming that floral and nesting resources will be suitable, we expect many bee species to extend their ranges to higher elevations on the San Francisco Peaks. However, it is likely that global warming could also reduce habitat suitability for key high elevation bee taxa (Wilson et al., 2005), such as *Bombus* and Megachilidae, and lead to replacement by other low elevation bee taxa. We have found that in this community along the elevation gradient bee communities have a higher turnover in species along the gradient than flies (McCabe et al., 2019).

Understanding the nature of environmental filtering at high elevations can help predict how these communities will change in the future. Although we focused on temperature as a limiting factor for bees at high elevations, other factors such as precipitation, floral resources (Lefebvre et al., 2018), canopy cover (McCabe et al., 2019), accepted with revisions), hypoxia (Dillon, Frazier & Dudley, 2006), air density (Dillon & Dudley, 2014) and competition (Pyke, 1982) must also be assessed when looking at how species can change along an elevation gradient. A companion study documented the importance of increasing forest cover as a factor limiting bees but not flies with increasing elevation (McCabe et al., 2019). There may be other important factors such as floral host plants and nesting resources (i.e., trees) in addition to abiotic constrains such as temperature. Insect distribution can rely heavily on the plant distribution, especially bees with strong ties to host plants (Schaffers et al., 2008; Siemann et al., 1998). Additionally, Kemp & Ellis (2017) found that insect community assemblage was based more on plant community assemblage than abiotic factors such as temperature. Overall, we have found that temperature may influence a reduction in bee diversity and abundance in high elevation communities, providing at least one factor to explain the bee to fly transition along an elevation gradient.

## Conclusion

Our research supports the notion that bee species, in general, are potentially more restricted by colder temperatures than flies, which in the past has been primarily attributed to differences in life history traits between these two taxa. In general, insects have adapted to colder temperatures by becoming larger and darker (Hagen et al., 2007; Hoiss et al., 2012; Wilson et al., 2007). Here we have shown that high elevation bees are likely to be more vulnerable than bee species that specialize in lower elevation habitats or pollinating fly species, due to their already specialized population to colder temperatures habitats. Assessing winners and losers with climate change is critical for understanding the evolutionary dynamics of bees, pollinator communities, and plant-insect interactions that will emerge during this century.

##  Supplemental Information

10.7717/peerj.7867/supp-1 File S1 Bee and Fly volume and darkness raw data Raw data measurements of bee and fly volume and darkness.

## Additional Information and Declarations

### Field Study Permissions

The following information was supplied relating to field study approvals (i.e., approving body and any reference numbers): Field collection was approved by the US Forest Service (NO. 0596-0082).

### Data Availability

The following information was supplied regarding data availability: The raw data measurements are available in a Supplemental File.

## References

[ref-1] Cane JH, Griswold T, Parker FD (2007). Substrates and materials used for nesting by North American Osmia bees (Hymenoptera: Apiformes: Megachilidae). Annals of the Entomological Society of America.

[ref-2] Cane JH, Minckley RL, Kervin LJ (2001). Sampling bees (Hymenoptera: Apiformes) for pollinator community studies: pitfalls of pan-trapping. Journal of the Kansas Entomological Society.

[ref-3] Classen A, Steffan-Dewenter I, Kindeketa WJ, Peters MK (2017). Integrating intraspecific variation in community ecology unifies theories on body size shifts along climatic gradients. Functional Ecology.

[ref-4] Cornwell WK, Ackerly DD (2009). Community assembly and shifts in plant trait distributions across an environmental gradient in coastal California. Ecological Monographs.

[ref-5] Coyle JR, Halliday FW, Lopez BE, Palmquist KA, Wilfahrt PA, Hurlbert AH (2014). Using trait and phylogenetic diversity to evaluate the generality of the stress-dominance hypothesis in eastern North American tree communities. Ecography.

[ref-6] Dillon ME, Dudley R (2014). Surpassing Mt, Everest: extreme flight performance of alpine bumble-bees. Biology Letters.

[ref-7] Dillon ME, Frazier MR, Dudley R (2006). Into thin air: physiology and evolution of alpine insects. Integrative and Comparative Biology.

[ref-8] Goslee SC, Urban DL (2007). The ecodist package for dissimilarity-based analysis of ecological data. Journal of Statistical Software.

[ref-9] Hagen BS, Jepsen JU, Ims RA, Yoccoz NG (2007). Shifting altitudinal distribution of outbreak zones of winter moth Operophtera brumata in sub-arctic birch forest: a response to recent climate warming?. Ecography.

[ref-10] Haslett JR (1989). Adult feeding by holometabolous insects: pollen and nectar as complementary nutrient sources for Rhingia campestris (Diptera: Syrphidae). Oecologia.

[ref-11] Heinrich B (1974). Thermoregulation in Endothermic Insects. Science.

[ref-12] Heinrich B (1975). Energetics of pollination. Annual Review of Ecology and Systematics.

[ref-13] Heinrich B (1996). The Thermal Warriors: Strategies of Insect Survival.

[ref-14] Hodkinson ID (2005). Terrestrial insects along elevation gradients: species and community responses to altitude. Biological Reviews.

[ref-15] Hoiss B, Krauss J, Potts SG, Roberts S, Steffan-Dewenter I (2012). Altitude acts as an environmental filter on phylogenetic composition, traits and diversity in bee communities. Proceedings of the Royal Society B: Biological Sciences.

[ref-16] Inouye D, Pike G (1988). Pollination biology in the Snowy Mountians of Australia: comparison with montane Colorado. USA. Austrailan Journal of Ecology.

[ref-17] Kearns C (1992). Anthophilous fly distribution across an elevation gradient. American Midland Naturalist.

[ref-18] Keller F, Kienast F, Beniston M (2000). Evidence of response of vegetation to environmental change on high-elevation sites in the Swiss Alps. Regional Environmental Change.

[ref-19] Kemp JE, Ellis AG (2017). Significant local-scale plant-insect species richness relationship independent of abiotic effects in the temperate Cape Floristic Region biodiversity hotspot. PLOS ONE.

[ref-20] Kingsolver JG (1983). Ecological significance of flight activity in Colias butterflies: implications for reproductive strategy and population structure. Ecology.

[ref-21] Körner C (2003). Alpine plant life: functional plant ecology of high mountain ecosystems; with 47 tables.

[ref-22] Kraft NJ, Ackerly DD (2010). Functional trait and phylogenetic tests of community assembly across spatial scales in an Amazonian forest. Ecological Monographs.

[ref-23] Laliberté E, Legendre P, Shipley B (2014). https://cran.r-project.org/web/packages/FD/FD.pdf.

[ref-24] Larson BMH, Kevan PG, Inouye D (2001). Flies and flowers: taxonomic diversity of anthophiles and pollinators. The Canadian Entomologist.

[ref-25] Lavorel S, Garnier E (2002). Predicting changes in community composition and ecosystem functioning from plant traits: revisiting the Holy Grail. Functional Ecology.

[ref-26] Lee R (2012). Insects at low temperature.

[ref-27] Lefebvre V, Villemant C, Fontaine C, Daugeron C (2018). Altitudinal, temporal and trophic partitioning of flower-visitors in Alpine communities. Scientific Reports.

[ref-28] Mayr E (1956). Geographical character gradients and climatic adaptation. Evolution.

[ref-29] McCabe LM, Colella E, Chesshire P, Smith D, Cobb NS (2019). The transition from bee-to-fly dominated communities with increasing elevation and greater forest canopy cover. PLOS ONE.

[ref-30] McCall C, Primack RB (1992). Influence of flower characteristics. weather, time of day, and season on insect visitation rates in three plant communities. American Journal of Botany.

[ref-31] Merriam C (1890). Results of a biological survey of the San Francisco Mountain region and the desert of the Little Colorado, Arizona. North American Fauna.

[ref-32] Messier J, McGill BJ, Lechowicz MJ (2010). How do traits vary across ecological scales? A case for trait-based ecology. Ecology Letters.

[ref-33] Michener CD (2007). The bees of the World.

[ref-34] Oksanen J, Blanchet FG, Friendly M, Kindt R, Legendre P, McGlinn D, Minchin PR, O’Hara RB, Simpson GL, Solymos P, Henry M, Stevens H, Szoecs E, Wagner H (2017). http://cran.r-project.org.

[ref-35] Parmesan C (2006). Ecological and evolutionary responses to recent climate change. Annual Review of Ecology, Evolution, and Systematics.

[ref-36] Peat J, Darvill B, Ellis J, Goulson D (2005). Effects of climate on intra-and interspecific size variation in bumble-bees. Functional Ecology.

[ref-37] Primack R (1978). Variability in New Zealand montane and alpine pollinator assemblages. New Zealand Journal of Ecology.

[ref-38] Pyke GH (1982). Local geographic distributions of bumblebees near Crested Butte. Colorado: competition and community structure. Ecology.

[ref-39] Rahbek C (1995). The elevational gradient of species richness: a uniform pattern?. Ecography.

[ref-40] R Core Team (2013). http://www.R-project.org/.

[ref-41] Schaffers AP, Raemakers IP, Sýkora KV, Ter Braak CJ (2008). Arthropod assemblages are best predicted by plant species composition. Ecology.

[ref-42] Siemann E, Tilman D, Haarstad J, Ritchie M (1998). Experimental tests of the dependence of arthropod diversity on plant diversity. The American Naturalist.

[ref-43] Smith RD, Higgins J, Burton J, Cobb NS (2015). Bee diversity and abundance along an elevational gradient in Northern Arizona. The Colorado Plateau VI: Science and Management at the Landscape Scale.

[ref-44] Stevens GC (1992). The elevational gradient in altitudinal range: an extension of Rapoport’s latitudinal rule to altitude. The American Naturalist.

[ref-45] Stone GN, Willmer PG (1989). Warm-Up Rates and Body Temperatures in Bees: The Importance of Body Size. Thermal Regime and Phylogeny. Journal of Experimental Biology.

[ref-46] Terborgh J (1977). Bird species diversity on an andean elevational gradient. Ecology.

[ref-47] Triplehorn CA, Johnson NF (2005). Borror and DeLong’s introduction to the study of insects.

[ref-48] Verdú JR, Lobo JM (2008). Ecophysiology of thermoregulation in endothermic dung beetles: ecological and geographical implications. Insect Ecology and Conservation.

[ref-49] Wilson RJ, Gutiérrez D, Gutiérrez J, Martínez D, Agudo R, Monserrat VJ (2005). Changes to the elevational limits and extent of species ranges associated with climate change. Ecology Letters.

[ref-50] Wilson RJ, Gutierrez D, Gutierrez J, Monserrat VJ (2007). An elevational shift in butterfly species richness and composition accompanying recent climate change. Global Change Biology.

[ref-51] Wu Z, Dijkstra P, Koch GW, Hungate BA (2012). Biogeochemical and ecological feedbacks in grassland responses to warming. Nature Climate Change.

[ref-52] Zeng J, Zhao D, Li H, Huang R, Wang J, Wu QL (2016). A monotonically declining elevational pattern of bacterial diversity in freshwater lake sediments. Environmental Microbiology.

